# “Until people start dying in droves, no actions will be taken”: perception and experience of HIV-preventive measures among people who inject drugs in northwestern Russia

**DOI:** 10.1186/s12954-017-0162-1

**Published:** 2017-06-05

**Authors:** Peter Meylakhs, Aadne Aasland, Arne Grønningsæter

**Affiliations:** 1 International Centre for Health Economics, Management and Policy, National Research University—Higher School of Economics, 194100 Kantemirovstaya st. 3a, St. Petersburg, Russia; 2 Norwegian Institute for Urban and Regional Research (NIBR), Oslo and Akershus University College of Applied Sciences, Norway, Holbergs gate 1, 0166 Oslo, Norway; 3 Fafo Institute for Labour and Social Research in Oslo, Norway, Borggata 2B, 0608 Oslo, Norway

**Keywords:** HIV prevention, People who inject drugs (PWID), Qualitative research, Russia

## Abstract

**Background:**

The HIV epidemic among people who inject drugs (PWID) in Russia continues to spread. This exploratory study examines how HIV-prevention measures are perceived and experienced by PWID in the northwestern region of Russia.

**Methods:**

Purposive sampling was used to obtain a variety of cases that could reflect possible differences in perception and experience of HIV-prevention efforts. We conducted 22 semi-structured interviews with PWID residing in the Arkhangelsk and St. Petersburg regions.

**Results:**

The main sources of prevention information on HIV for PWID were media campaigns directed to the general population. These campaigns were effective with regard to communicating general knowledge on HIV but were ineffective in terms of risk behavior change. The subjects generally had trust in medical professionals and their advice but did not follow prevention recommendations. Most informants had no or very little prior contact with harm reduction services. On the level of attitudes towards HIV prevention efforts, we discovered three types of fatalism among PWID: “personal fatalism” - uselessness of HIV prevention efforts, if one uses drugs; “prevention-related fatalism” - prevention programs are low effective, because people do not pay attention to them before they get infected; “state-related fatalism” – the lack of belief that the state is concerned with HIV prevention issues. Despite this fatalism the participants opined that NGOs would do a better job than the state as they are “really working” with risk groups.

**Conclusions:**

As HIV prevention campaigns targeted at the general population and prevention advice received from medical professionals are not sufficiently effective for PWID in terms of risk behavior change, prevention programs, such as community-based and peer-based interventions specifically tailored to the needs of PWID are needed, which can be achieved by a large expansion of harm reduction services in the region. Personal communication should be a crucial element in such interventions in addition to harm reduction materials provision. Training programs, peer outreach, and culture-change interventions which try to alter widespread fatalistic norms or attitudes towards their health are especially needed, since this study indicates that fatalism is a major barrier for behavior change.

## Background

Russia has one of the fastest-growing HIV/AIDS epidemics in the world, driven primarily by the spread of infection among people who inject drugs (PWIDs). HIV incidence is increasing in the general population—in 2013 there were 79,728 new HIV cases registered in the Russian Federation, compared to 58,142 in 2009 [1], and among PWIDs, where the incidence of HIV in some regions is as high as 7.2/100 person-years [2].

From the onset of the HIV epidemic in Russia, the authorities concentrated their preventive efforts on the general population (through media campaigns, etc.) paying almost no attention to PWID. Any preventive efforts undertaken among PWID were carried out mainly by NGOs funded by foreign organizations dealing with HIV/AIDS. However, despite the lack of involvement in preventive work with PWID—and thus with no supporting evidence—in 2008, the Russian authorities dismissed such work as “useless” and declared that they would focus on “promoting a healthy lifestyle” instead. That was done in total disregard of advice from international institutions like the WHO and UNAIDS that recommended targeted preventive efforts among PWIDs (syringe exchange programs, substitution therapy, peer-to-peer work, etc.) as highly effective in curtailing the spread of HIV [3–6].

This exploratory study seeks to give a voice to one of the most neglected groups in Russian society—people who inject drugs—and to see how they themselves perceive federal and local HIV-prevention efforts. We explore a range of issues relevant for HIV prevention among PWID, including how information on prevention is communicated, sources of such information, attitudes to and trust in this information, and whether the advice is followed.

## Methods

### Sampling and recruitment

As this study is of exploratory character, we used purposive sampling to get the fullest picture possible within the limitations of the study, that is, to obtain a variety of cases that could reflect possible differences in perception and experience of HIV-prevention efforts. We chose two regions, which were very different from each other in terms of the HIV epidemic level. We chose Arkhangelsk region, which is characterized by a low level of the epidemic (42.6 per 100,000 people, according to official statistics) and St. Petersburg region where the prevalence of HIV is among the highest in Russia—996.5 per 100,000 people (compared to the national level of 428.8 per 100,000 people) [1]. Within each region, we have chosen two sites—a big city and a small town. In both Arkhangelsk and in St. Petersburg, there were syringe exchange services available to PWID; however, their number was miniscule compared to this key population needs. In Arkhangelsk, there was only one service where users could exchange syringes (data on coverage is unavailable). In St. Petersburg, there were three syringe-exchange programs. The largest of them provided by NGO “Humanitarian Action” served 2500 people a year at the time of this study execution [7]. Given that the number of PWID in St. Petersburg at that time was estimated to be around 80,000 [8] one can see how limited the coverage was (and still is). There were no harm reduction services in small towns.

The sample consisted of 22 participants, who reported having injected drugs at least once in the week prior to the interview. The sample’s characteristics are presented in Table 1.

**Table 1:** Sociodemographic characteristics and HIV status of study participants

Arkhangelsk region							St. Petersburg region						
Location		Gender		HIV status			Location		Gender		HIV status		
Town	City	Female	Male	Unknown	HIV+	HIV-	Town	City	Female	Male	Unknown	HIV+	HIV-
2	4	3	3	2	1	3	6	10	7	9	1	10	5

Study participants ranged in age from 25 to 37 years, with an average age of 32. The number of years since participants began injecting ranged from 9 to 21, with an average of 14 years. Heroin was the drug of choice for all participants, all of whom reported physical dependence on heroin at the time of the interview.

To recruit participants, the researchers established contacts with a harm-reduction NGO in each region that provided services for PWID and sex-workers, the City Drug Treatment Center, and the City AIDS Center. Each venue assigned a person to help researchers recruit subjects for the study—an outreach worker in the harm-reduction NGO, and social workers at the City Drug Treatment Center and the City AIDS Center. Study goals and eligibility criteria for participants (injecting drugs at least once a week prior to the interview) were explained to the recruiters. They were instructed to seek potential informants who were diverse in terms of age, gender, HIV status. After having obtained preliminary consent to participate from a potential informant, recruiters informed the researcher responsible for the fieldwork (PM); if the potential informant was found of interest for the study, the recruiter referred the participant to the researcher or conducted the interview him/herself, depending on practical circumstances. Study participants were recruited from a range of venues: harm-reduction centers, drug treatment centers, AIDS centers as well as “from the street” by harm-reduction outreach workers. Recruitment proved difficult in small towns, as there were no services for PWID; in Arkhangelsk region the street outreach workers exploited their social networks fully, but still managed to recruit only two participants (see Table 1). Budgetary and organizational constraints forced us to stop the recruitment process after 5 months, March to July 2009.

### Data collection

Data collection, consisting of semi-structured interviews, was conducted in 2009. Since then, little has changed in the Russian approach to HIV-prevention among PWID, so it is likely that results and conclusions of this study are still valid.

The interview guide’s topics included: informants’ backgrounds and their histories of drug use and/or HIV-infections; perception and experience of HIV-prevention measures; doctor-patient relationship (for HIV-positives); trust in prevention recommendations; following prevention recommendations, perception of challenges to HIV-prevention, informants’ views on how preventive work with vulnerable groups should be organized. Interviews were conducted by researchers, social workers, and outreach workers. Social workers attended a workshop on qualitative data collection. First author (PM) instructed outreach workers on how to collect a semi-structured interview and discussed the project guide with them.

Based on their individual preferences, participants were interviewed on the street, in their homes, semi-public places (cafes and diners), or private rooms at the participating services. Interviews were tape-recorded and conducted in Russian language and lasted on average about 1.5 h. All subjects signed the informed consent and were reimbursed for their time and effort with the sum in rubles equal to US$ 30. The study was approved by the Review Board of the Research Council of Norway.

Data (or theoretical) saturation is an important criterion in qualitative research that bears on validity of the study results [9]. It refers to the stage at the data collection and analysis process where recruitment of additional participants stops bringing new information or insights with regard to the study’s findings and concepts. Although there are no clear-cut standards on data saturation (in terms of the study’s conceptual elaboration or the number of subjects needed to achieve it) [10], we are reasonably confident that we achieved data saturation regarding the main findings reported in this study, such as our participants’ fatalism with regards to prevention efforts and their own health, and other findings. While the number of participants from small towns is clearly insufficient for making a claim that we have captured the experience of and attitude to HIV prevention of small town PWID (especially in Arkhangelsk region – only 2 participants), we report no separate findings concerning the lives of small town PWID apart from a pretty apparent one, the lack of NGOs dealing with HIV in these areas. Clearly, further research is needed to examine the complexities of lives of this population, including those that are related with HIV prevention.

### Analysis

Participant interviews were transcribed verbatim. The transcripts were translated into the English language by two Russian-speaking members of a major international HIV advocacy organization with advanced competency in English. One member of the research team, who has command of both Russian and English (Russian being his mother tongue), has checked the validity of translations. The translated transcripts were entered into Open Code qualitative software for analysis.

A grounded theory approach was used for data analysis [11]. An initial list of broad categories to be used for coding was developed, prior to the interviews based on the logic of the study, researchers’ prior experience, and literature in the field. Thus, for example, categories such as sources of prevention information and trust in prevention information were of obvious relevance for the study. Each category was assigned a set of corresponding codes. The coding proceeded in inductive and iterative fashion in several iterations, during which new codes were introduced with a purpose to capture emergent concepts that had not figured at the previous stages of analysis. The process was accompanied by extensive theoretical memo-writing, which is a key component of grounded theory analysis [9], aimed at further developing existing categories, producing new ones, and exploring relationships among categories. Thus, we proceeded from purely descriptive categories to analytical ones (e.g., fatalism), whereupon we continued our analysis until we elaborated different conceptual dimensions of the categories used in the analysis and examined relationships among them.

## Results

### Perception of HIV preventive measures

Informants from Arkhangelsk region (low-level of HIV infection) and St. Petersburg region (high-level of HIV infection) had virtually identical attitudes to and experience with HIV prevention efforts. However, there was one difference between cities and small towns. Informants from big cities named NGOs dealing with HIV/AIDS as sources of preventive information, while informants residing in small towns did not mention such NGOs (while the way the informants were recruited may have influenced the observed differences as NGOs were utilized for recruitment as was described in the Methods section, these differences cannot be attributed only to this fact—in large cities most of the informants that were recruited through other venues (e.g., The Drug Treatment Center) had heard about organizations that were helping drug users, while no one from small towns had heard about such organizations). The sources named by PWID were media, public campaigns (newspapers, TV, radio, brochures, posters in clinics); peers; and school programs (elements of sex education).

Perceptions of the HIV prevention media campaigns were somewhat contradictory. For some, media campaigns were “dull” and they felt they did not concern them. Others described them as fear campaigns, especially early media campaigns, when HIV was portrayed as a “plague” with no chance of survival. Many informants avoided watching them so as not to “spoil the mood.”

*‘…there was one poster at the office of an infectious diseases doctor, we were examined there; so there was an enormous HIV poster, and there were insects crossed with a red cross, to show they were not carriers, and many, many other things. And it was terrible, just terrible. I cannot say that I read the information because some pictures were so scary to me that I did not want to read about that’* (f, 29, Arkhangelsk region).

Several informants expressed pessimism about the utility of prevention information because people do not pay attention to it unless they get HIV. Especially, such an attitude (which can be called “prevention-related fatalism”) was characteristic of HIV-infected informants, who grounded their opinion in their own experience.

*‘R: I think that there are enough posters and there, on these posters, there are links to HIV sites. No point in prevention before a person gets sick.*

*I: And would you add anything new?*

*R: What for? When a person gets sick, gets HIV status, then it’s possible to talk about it.’* (m, 32, St. Petersburg region).

Also, several informants underscored that personal example and personal communication are far more effective than merely providing information, as people are more receptive to the specific experiences of others. A syringe-exchange program worker told us that handing out brochures containing prevention information was useless: clients do not read them, because they come with specific purposes, like syringe exchange or consultation. In her view, personal communication is much more effective.

Apart from information campaigns, none of our respondents had any experience of other governmental prevention measures. However, almost all our subjects thought that the state had the potential to organize good prevention policy, but that state policy seemed not to support this. As one informant grimly expressed his pessimism, relating his personal experience to indifference on the part of the state indifference:

*‘…it seems to me that as far as people are not affected by this problem, family, someone else, then people will not do anything. I can relate to my own experience, before I was affected, I didn’t care … Well, I think people of power are as far from this problem as I was. And until people will die in droves from all this, no actions will be taken. If something is done, it is done by non-governmental organizations, and some foundations. It is necessary to try to combat this!’* (m, 25, St. Petersburg)

The belief that the state has the capacities but lacks the political will to alleviate the HIV-epidemic can be called “state-related fatalism.” This attitude towards the state stands in stark contrast to respondents’ attitudes towards the NGOs that deal with HIV issues. It is particularly noteworthy because most our respondents had little or no prior contact with harm-reduction services. Nevertheless, they underscored the need for “special programs” for vulnerable groups to be organized on a large-scale, such as syringe exchange programs. They felt that NGOs could do a better job than the state, as they are “really working” with risk groups. The feeling that “no one cares about them” except for NGOs is echoed in earlier studies on PWID in Russia [12]. Here, another difference emerges between big cities and small towns concerning HIV prevention, as PWID living in small towns are particularly disadvantaged regarding the availability of HIV services and NGO presence.

While mass education campaigns providing information on transmission routes of HIV must be said to have been successful since all our respondents knew the facts, this information is seen as being of little practical use: study participants considered it either too frightening to read/ watch, or too boring. One participant describes his experience of watching a short AIDS-prevention documentary in school:

*‘Well, just description of HIV, how it is transmitted and where it came from. That’s all. Nothing interesting.’* (m, 28, St. Petersburg region).

None of our informants indicated that the information received through mass education campaigns motivated them to change their risk practices.

### Trust in prevention information and following prevention advice

We found general trust in all the above-mentioned sources of prevention information among PWID, as well as trust in medical professionals and their advice. While trust in doctor-patient relationships has been shown to be important in health care utilization, patient activation, and treatment outcomes [13–15], and some studies suggest that it also plays a positive role in reducing PWID risk practices—[16] in our study (given its limitations), we could not confirm that our informants’ trust in their doctor reduces their risk practices.

Russian PWID live in a very high-risk environment [17, 18] illegal status of the substances they use, permanent police harassment, insufficient number of 24/7 pharmacies where one can buy a clean syringe, and no or negligible presence of SEPs. These structural factors, coupled with their drug dependency, make it very hard to follow preventive advice. PWID have to manage a whole “hierarchy of risks,” such as risk of being in withdrawal, risk of police arrest etc., so risk of contracting a blood-borne virus may not rank very high on some PWID “risk agenda” [19].

This may explain why only around one-fourth of our subjects said they did not share syringes. Some of them, who are now HIV-positive, “just didn’t think it would happen to them.” For others, including those who were HIV-negative, it was a non-caring attitude towards their health.

*‘If we speak about unprotected sex – I had just complete indifference. I mean apathy towards myself and, naturally, to other people. It’s true. And if we speak about instruments for injections – it’s the same. The same indifference. Because when I wanted to get high, I did not care how and with which kind of syringe, I did not care. That’s it.’* (m, 27, St. Petersburg region).

We can call such an attitude fatalistic as informants see no point in taking care of their health because they use drugs and leave what will happen to them to chance.

Here is an even more telling example illustrating this point:

*‘Well, I know how. Well, we went with a girl, we were in need of using drugs, she said she had HIV, “you want – take it, you don’t want - do not take a syringe after me”. I took her syringe, I'm sure I got infected then. There was some blood, I had nothing to wash it with, there was nothing. Well, that’s it.’* (f, 28, St. Petersburg region)

Others explained sharing needles in terms of mistrust of their co-injectors—they did not have their own syringes but did not want to leave the others and go to get one, because they were sure that no drugs would be left when they got back—or there were no pharmacies in the vicinity.

This finding is in agreement with other studies, which show fatalistic attitudes among many PWID to blood-borne infections such as HCV and/or HIV [20]. In our view, this fatalism is at least partly attributable to popular and medical discourses about drug addiction that underscore the powerlessness of an addicted person to control life circumstances (including risk situations). The almost total absence of services for PWID may be another source of this fatalism.

Before proceeding to the discussion section we would like to make an important caveat: It may well be that for some HIV-positive informants personal fatalism that was revealed in this study is a psychological artifact—a psychological defense mechanism that these people used to come to terms with their diagnosis, and not a world-view that determined their risk practices. However, fatalistic attitude to life was often found among HIV-negative informants; therefore, we can reasonably assume that personal fatalism was one of the factors that contributed to our informants’ risk practices.

## Discussion

We identified three types of fatalism among our informants. “Personal fatalism” is expressed in the attitude that there is no point in taking care of your health if you use drugs anyway. As the structural position of PWID is very precarious and rife with uncertainties (stigma, harassment, strict laws, illness/death of their friends) [21, 22], it seems plausible to see this position as contributing to fatalistic attitudes towards their own health.

Second, “prevention-related fatalism” involves the perception that prevention programs are not effective because people do not pay attention to them unless they get infected.

The third type may be called “state-related fatalism”: the unwillingness of the state to solve HIV-prevention issues “until people start dying in droves.”

It can be argued that the three types of fatalism are interrelated. Personal fatalism can lead to prevention-related fatalism: when PWID observe fatalistic attitude in a large number of their friends and acquaintances, who inject drugs, they can come to a conclusion that any efforts to change drug injectors’ risk behaviors are futile. On the other hand, the negligible presence of services tailored for PWID’s needs may generate the feeling of being abandoned by the state—state-related fatalism—and, as was mentioned above, also contribute to personal fatalism. The belief that the state is unwilling to intervene decisively despite the growing number of new HIV infections among PWID (state-related fatalism) may lead to prevention-related fatalism, especially if the state is expected to be the leading actor in social problems solving (which is often the case in post-Soviet countries [23]).

We believe that the notion of “fatalism”, and, in particular, “personal fatalism” is a useful concept that has an explanatory potential in trying to answer the question, why some PWID successfully avoid blood-borne infections while others fail to do so even in the settings where services for PWID are prevalent (e.g., London, New York City [20, 24]). Thus, in Meylakhs et al. [24] study on PWID, who stay safe in the long run in New York City, it was found that some PWID who got infected with HIV or HCV or both viruses tended to have a fatalistic attitude towards their health and life in general in contrast to those who successfully avoided infection with either virus. More research in diverse settings is needed to examine the concept of “fatalism”, namely: to what extent fatalistic attitudes towards one’s own health are influenced by personality traits and what these traits are, and to what extent is it produced by social and cultural factors such as the actions of the state or having a stable source of income? At what stage of a drug injector’s career is it formed and what factors facilitate its formation and, perhaps, later changes away from fatalism? What aspects of risk environments are conducive to the formation of fatalism? Last but not least, a methodological question arises: how to discern between “real” fatalism that constitutes a ground for action and “post-factum” fatalism—psychological rationalization that people produce as a coping mechanism for accepting their diagnosis, whereas prior to having been diagnosed they had not have a fatalistic attitude to their health? The answers to these and other questions could inform intervention programs directed to combat fatalism.

The study also revealed the two interrelated problems of communication about intervention programs specially tailored for PWID and the availability of such programs. Most of our informants did not have contacts with the services tailored for them, or had episodic encounters. Scarce availability of such programs renders regular contacts with such services very improbable. Communication and availability are thus interrelated: it is not particularly useful to tell people about programs that are not or hardly available; on the other hand, when the number of special services is small, it is hard to spread the word among the drug-using community via these channels.

## Conclusion

We would like to conclude this paper with the following recommendations based on this study:

Our informants’ perception of HIV prevention campaigns targeted at the general population indicates that these campaigns are insufficiently effective for PWID in terms of changing their risk practices. Therefore, as many other researchers stress [25–27] urgent scaling up of harm reduction programs should be a top HIV-prevention priority in Russia. Coverage should be substantial, otherwise it will not affect the epidemiological situation with HIV. And only significant and visible presence of harm reduction programs would break the vicious circle of nonavailability of harm reduction programs and spreading the word in the community about them.

Expansion of community-based and peer-based interventions are needed as the study data indicate that even good and trustful relations with medical professionals are insufficient for changing risk behaviors. Our study also demonstrates that personal communication should be a crucial element in such interventions (aside from mere harm reduction material provision). Ideally these interventions should be oriented towards creation of norms of non-sharing syringes and other drug paraphernalia among PWID. A number of studies have shown that PWID can be effective agents in risk reduction (e.g., [28]). What Friedman et al. [29] call “intraventions,” that is, “prevention activities that are conducted and sustained through processes within communities themselves” ([29]: 251) can be an important addition to risk reduction intervention programs implemented by public health professionals.

Training programs and outreach work with PWID combating their fatalistic attitude towards their health are especially needed as the results of this study indicate that it is a major psychological barrier for behavior change.

### Study limitations

The main limitation of this study is a small number of respondents residing in small towns. Self-reported status of HIV is another limitation; especially, we cannot be certain that those informants that reported no HIV-infection are indeed non-infected. Recall problems is yet another limitation as the informants remembered events that had happened long before the interviews were taken. In attributing to Arkhangelsk and St. Petersburg regions low-prevalence and high-prevalence statuses, respectively, we relied on the official data that reflects the number of registered users only.
